# Targeting Therapeutic Antibodies to the CNS: a Comparative Study of Intrathecal, Intravenous, and Subcutaneous Anti-Nogo A Antibody Treatment after Stroke in Rats

**DOI:** 10.1007/s13311-020-00864-z

**Published:** 2020-05-06

**Authors:** Anna-Sophia Wahl, Daphne Correa, Stefan Imobersteg, Michael Andreas Maurer, Julia Kaiser, Marc Aurel Augath, Martin E. Schwab

**Affiliations:** 1 Brain Research Institute, University of Zurich, Winterthurerstr. 190, 8057 Zurich, Switzerland; 2 Central Institute of Mental Health, University of Heidelberg, J5, 68159 Mannheim, Germany; 3 Department of Health Sciences and Technology, ETH Zurich, Universitätsstrasse 2, 8092 Zurich, Switzerland; 4 Institute for Regenerative Medicine (IREM), University of Zurich, Wagistrasse 12, 8952 Zurich, Switzerland; 5 Institute for Biomedical Imaging, ETH Zurich, Wolfgang-Pauli-Strasse 27, 8093 Zürich, Switzerland

**Keywords:** CNS antibody delivery, stroke, intrathecal, functional recovery, Anti-Nogo antibody therapy

## Abstract

Antibody-based therapeutics targeting CNS antigens emerge as promising treatments in neurology. However, access to the CNS is limited by the blood–brain barrier. We examined the effects of a neurite growth-enhancing anti-Nogo A antibody therapy following 3 routes of administration—intrathecal (i.t.), intravenous (i.v.), and subcutaneous (s.c.)—after large photothrombotic strokes in adult rats. Intrathecal treatment of full-length IgG anti-Nogo A antibodies enhanced recovery of the grasping function, but intravenous or subcutaneous administration had no detectable effect in spite of large amounts of antibodies in the peripheral circulation. Thus, in contrast to intravenous and subcutaneous delivery, intrathecal administration is an effective and reliable way to target CNS antigens. Our data reveal that antibody delivery to the CNS is far from trivial. While intrathecal application is feasible and guarantees defined antibody doses in the effective range for a biological function, the identification and establishment of easier routes of administration remains an important task to facilitate antibody-based future therapies of CNS disorders.

**Electronic supplementary material:**

The online version of this article (10.1007/s13311-020-00864-z) contains supplementary material, which is available to authorized users.

## Introduction

Antibodies targeting CNS antigens were shown to be promising novel therapeutic tools for diseases like Alzheimer’s and Parkinson’s diseases, multiple sclerosis, stroke, and spinal cord injury [1]. The route of application in recent clinical trials was mostly intravenous (i.v.). Due to the blood–brain barrier, very low proportions of antibodies reach the CNS parenchyma. To overcome this biological barrier, very large doses of antibodies were applied in these studies (up to grams per injection [2, 3]). The negative outcome of several recent clinical trials, e.g., in Alzheimer’s disease, may well in part be due to insufficient amounts of therapeutic antibodies reaching the CNS.

Intrathecally or intracerebroventricularly applied, function-blocking antibodies against the neurite growth-inhibiting and plasticity-restricting membrane protein Nogo A and its receptors have been shown in various laboratories in different stroke and spinal cord injury animal models to enhance regenerative and compensatory sprouting as well as functional recovery in rodents and primates [4, 5]. A phase I clinical trial in spinal cord-injured patients has shown intrathecal human anti-human Nogo A antibody injection or infusion over 30 days to be safe [4]. A randomized double-blind placebo-controlled phase II clinical trial is ongoing (https://clinicaltrials.gov/ct2/show/NCT03935321). However, for broader clinical applications, e.g., in stroke patients, a systemic rather than an intrathecal application would be preferable. The same is true for other therapeutic antibodies and neurological indications, especially if long-term treatments in outpatients are required. Here, we compare intrathecal, intravenous, and subcutaneous administrations of a therapeutic antibody against the neurite growth inhibitor Nogo A in adult rats with large forebrain strokes in their ability to restore forelimb fine motor function.

## Methods

### Animals

A total of 57 adult female Long–Evans rats (200-250 g, 12-16 weeks old, Janvier, Le Genest-Saint-Isle, France) were included. The animals were housed in groups of 2 to 4 in open airflow cages under a 12-h dark/light cycle. Food and water were delivered *ad libitum* except for restricted periods during the single-pellet grasping and staircase training and testing phases. All experimental procedures were approved by the Veterinary Office of the canton of Zurich, Switzerland.

### Experimental Setup

**Fig. 1:**
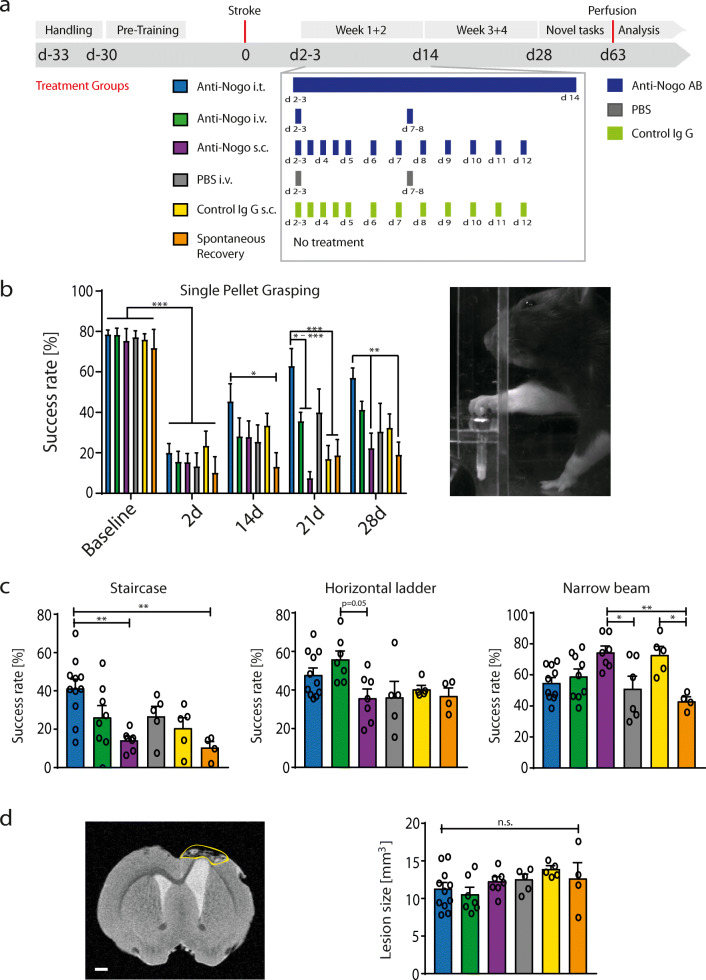
Intrathecal anti-Nogo application promotes functional recovery after stroke while intravenous and subcutaneous application routes fail. (A) Experimental timeline for the 6 experimental groups (“anti-Nogo i.t.,” *n* = 11; “anti-Nogo i.v.,” *n* = 8; “anti-Nogo s.c.,” *n* = 7; “PBS i.v.,” *n* = 6; “control IgG s.c.,” *n* = 5; “spontaneous recovery,” *n* = 4). The graph in particular depicts the frequency of the anti-Nogo antibody dosage through different routes of application (i.t., i.v., and s.c.) early after photothrombotic stroke in rats (treatment window 2-3 days till 14 days after insult). (B) Success rates in the single-pellet grasping task: Only the animals which had received anti-Nogo antibodies intrathecally (“anti-Nogo i.t.” group) showed a significant recovery 3 to 4 weeks after stroke. (C) The animals in the “anti-Nogo i.t.” group also performed better than all other groups in a second, novel grasping task (staircase test), introduced after the immunotherapy. The animals with the anti-Nogo intrathecal application in particular significantly exceeded the animals with subcutaneous application and those without treatment (“spontaneous recovery group”). The same effect could not be found for more general locomotion tasks such as the horizontal ladder and the narrow beam test. (D) Left: representative coronal MR image depicting the lesioned motor cortex in a rat brain 8 weeks after stroke, scale bar = 1000 μm. Right: stroke lesion size in all 6 treatment groups revealed no significant difference among groups. Data are presented as means ± SEM; statistical evaluation was carried out with 1-way (C, D) and 2-way ANOVA (B) repeated measure followed by Bonferroni *post hoc*; asterisks indicate significances: *P < 0.05, **P < 0.01, ***P < 0.001. n.s. = nonsignificant

All of the rats were first handled and then trained in the single-pellet grasping task as previously described [6, 7]. A large photothrombotic stroke [7] lesioned the sensorimotor cortex corresponding to the preferred paw of the animals in the grasping task. Only those animals with a significant lesion deficit (< 50% success rate in the grasping task compared to prestroke baseline levels) 2 days after stroke surgery and same lesion size and location (see below) were included (*n* = 41). The animals were then randomized into 6 different treatment groups (Fig. 1A): the animals in the “anti-Nogo i.t.” group received 8.4 mg/2 ml anti-Nogo antibody 11C7 [8] intrathecally via osmotic pump delivery into the lumbar CSF space until 14 days after stroke [6, 7]. In the “anti-Nogo i.v.” group, the animals were injected twice with 42 mg anti-Nogo antibodies (diluted in 5.6-6 ml PBS, depending on the antibody batch) over the course of 1 h into the femoral vein on days 2 or 3 and 7 to 8 after insult. Control animals received instead of antibodies intravenous injections of 5.6 to 6 ml PBS (“PBS i.v.” group). For the subcutaneous antibody application (“anti-Nogo s.c.” and “control IgG s.c.” groups), 1 ml of antibody solution (10 mg/ml anti-Nogo or control (FG12/B5) antibodies) was subcutaneously injected over the flank (alternating the side) starting on day 3 after stroke with 2 applications daily for days 2/3 to 5 (morning and evening with 12 h delay in between) followed by 1 injection per day up to day 12. Animals in the “spontaneous recovery” group received no treatment after stroke. To assess the degree of recovery of lost forelimb function, the animals were retested in the single-pellet grasping task on days 14, 21, and 28 after stroke. We also exposed the animals to novel (nontrained) tasks (sugar-pellet grasping in staircase, horizontal ladder, and narrow beam, as previously described [6, 7]), testing their overall functional recovery. Blood was taken to measure the antibody concentration on day 2/3, 7/8, 9/10, and 14 after stroke. For the intravenous and subcutaneous groups, blood was collected always before the next dose of antibody application on these days. Finally, the rats were transcardially perfused [6, 7].

To determine the tissue antibody concentration in the CNS, the animals either received 4.2 mg/ml anti-Nogo antibody 11C7 [8] intrathecally via continuous osmotic pump delivery into the lumbar CSF space (*n* = 5) starting on day 2 after stroke or were injected with 42 mg anti-Nogo antibodies over the course of 1 h into the femoral vein on day 2 after insult (*n* = 5). Both groups of animals were euthanized, CSF was taken from the cisterna magna, and the rats were perfused with ice-cold Ringer solution 7 days after stroke, followed by dissection and removal of brain and spinal cord tissues (lumbar spinal cord, thoracic spinal cord, cervical spinal cord, cerebellum, brainstem, stroke penumbra (1-2 mm around the stroke core), cortex (stroke side)). The tissues were immediately frozen at − 80° in liquid nitrogen for further tissue analysis with enzyme-linked immunosorbent assays (ELISAs) (see below). Except for *n* = 4 animals, CSF samples contained traces of blood and were thus not used for further analysis. All the animals were number-coded, and investigators responsible for the behavioral assessments, analysis, and interpretation of the data were blinded to the treatment groups until the end of the data analysis. This study followed the ARRIVE guidelines (https://www.nc3rs.org.uk/arrive-guidelines).

### Determination of Stroke Lesion Size

Lesion size and location were determined *ex vivo* in the perfused brains with a 7-T small animal MR system (Bruker BioSpin GmbH, Ettlingen, Germany) as previously described [7] on 20 T2-weighted frontal plane slices (thickness, 0.3 mm; interslice distance, 0.3 mm (no gap between slices)). The final calculations for the stroke lesion size were performed with Image J comparing the lesioned hemisphere to the intact one [7]. Only animals with a similar, large lesion size (> 8.0 mm^3^) and location destroying the sensorimotor cortex as determined by the MRI data were included (*n* = 41 of 47 rats).

### Detection of Infused Anti-Nogo A Abs in Serum, CSF, and CNS Tissue by ELISA

The tissue was homogenized in a weight-to-volume ratio of 1:3 in modified lysis buffer containing 200 mM sodium chloride, 20 mM Tris-HCl, pH 8.0, 1% NP-40, 1 mM EDTA, and protease inhibitors (Roche, Basel, Switzerland). The homogenate was centrifuged (16.000 rcf) to pellet the cell and tissue debris (maximally 15-25% of the total homogenate). The protein concentration in the supernatant (between 75 and 85% of the total homogenate) was measured with the RC-DC Protein Assay Kit (Bio-Rad, Hercules, CA). The concentration of anti-Nogo A antibodies in serum, CSF, and CNS tissue was measured with a sandwich enzyme-linked immunosorbent assay. Briefly, plates were coated with rabbit anti-mouse IgG (Invitrogen, Carlsbad, CA) as the primary antibody and blocked using 5% w/v skimmed milk powder in TBS-T buffer (Tris-buffered saline, 0.1% Tween 20) followed by incubation of samples. To detect the anti-Nogo A antibodies in samples, a HRP-conjugated goat anti-mouse IgG (Invitrogen) secondary antibody was used. The activity of horseradish peroxidase (HRP) was determined by a Pierce TMB ELISA Substrate kit (Thermo Scientific, Waltham, MA), and optical density of the HRP reaction was determined at 450 nm using a TECAN SPARK reader. The concentrations of anti-Nogo A antibodies in serum, CSF, and CNS tissue were then plotted against a standard curve of anti-Nogo A antibody.

### Statistical Analysis

Statistical evaluation was performed with Graph Pad Prism (GraphPad Software Inc.; Version 6.05). All data are expressed as means ± SEM. Group-dependent data with several time points were analyzed using 2-way repeated-measures ANOVA and Bonferroni’s *post hoc* test for the results of the single-pellet grasping. We chose a Bonferroni *post hoc* test due to the slightly unbalanced data set (different group sizes). One-way ANOVA repeated measures plus Bonferroni’s multiple-comparison tests was applied in the case of time-independent group values (lesion size analysis, novel tasks, and antibody concentrations comparing different tissues). The sample size for the different treatment groups was estimated by means and variance of measured data means in related work [6, 7, 9] predicted to be sufficient to detect a statistically significant result in ANOVA with *α* = 0.05 and power > 0.8. The level of significance was set at *P < 0.05, **P < 0.01, ***P < 0.001.

## Results

We compared an intrathecal anti-Nogo A antibody treatment over 2 weeks by lumbar pumps with intravenous (i.v.) and subcutaneous (s.c.) application routes with much larger quantities (10× for i.v., 15× for s.c.) to promote functional recovery after a large photothrombotic stroke in rats destroying the sensorimotor cortex (Fig. 1A). We found that precise grasping for sugar pellets through a window in the Plexiglas testing box (Fig. 1B) was destroyed in all groups 2 days after stroke (10-20% of baseline levels in all groups). The animals which received anti-Nogo antibodies (8.4 mg/2 ml) intrathecally, from day 2/3 to 14, via osmotic pumps revealed a significantly higher success rate in the pellet grasping task already 2 weeks after stroke compared to animals without treatment (“anti-Nogo i.t.” *vs* “spontaneous recovery” group, repeated-measures 2-way ANOVA with *post hoc* Bonferroni, *P* < 0.05, Fig. 1B). The animals with the intrathecal anti-Nogo treatment subsequently excelled compared to the other groups from 3 weeks onward (Fig. 1B). In contrast, the intravenous and subcutaneous applications of the anti-Nogo therapy failed to enhance the stroke-impaired grasping function compared to the control IgG, PBS, or untreated groups (Fig. 1B), although the applied anti-Nogo dosage was 10 times (for i.v.) and 15 times (for s.c.) higher than in the intrathecal condition. A tendency for improved recovery may be present in the intravenous anti-Nogo group, but results did not reach significance (in part due to the high variation inherent in these complex behavioral assays). The persistent low performance level of the “anti-Nogo s.c.” group was particularly remarkable, but correlated with the low serum levels of the antibodies (see below) (“anti-Nogo s.c.” *vs* “anti-Nogo i.t.” cohort 3 to 4 weeks after insult, repeated-measures 2-way ANOVA with *post hoc* Bonferroni, *P* < 0.01, Fig. 1B).

The animals with the intrathecal anti-Nogo application performed also best in another grasping task—the staircase test (Fig. 1C, success rate 41.3% ± 4.7 for the “anti-Nogo i.t.” animals *vs* 14.2% ± 1.9 for the “anti-Nogo s.c.” group *vs* 10.5% ± 3.0 for the “spontaneous recovery” group). As the animals were not trained in the staircase test, this result indicates a generalization of recovery for grasping function in the “anti-Nogo i.t.” group which is transferable to nontrained grasping tasks. However, when the animals were tested in 2 other novel tasks assessing general locomotion and balance—the horizontal ladder and the narrow beam—we did not find a specific effect of the anti-Nogo immunotherapy on the level of success compared to the control groups (Fig. 1C): although the animals with subcutaneous anti-Nogo treatment performed significantly better than the animals without treatment on the narrow beam (“anti-Nogo s.c. *vs* “spontaneous recovery” group, 1-way ANOVA with *post hoc* Bonferroni, *P* < 0.01, Fig. 1C), the same was true for the “IgG control s.c.” group. This result may point to a slightly compromised locomotion in animals with intrathecal and intravenous treatments due to scars and sutures from the implanted osmotic pump or the femoral vein injections. *Ex vivo* MRI at the end of the behavioral testing revealed the same stroke lesion size and location for all animals included in the analysis (Fig. 1D).

**Fig. 2:**
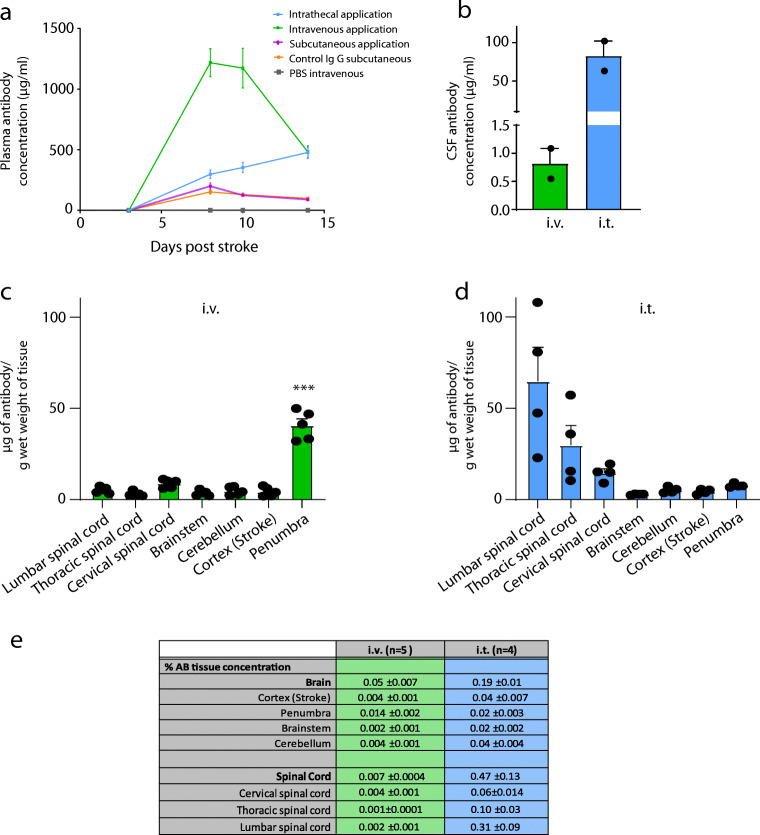
Plasma, CSF, and CNS tissue concentrations of anti-Nogo antibodies depending on the route of administration. (A) Plasma levels of anti-Nogo A antibody after intravenous (*n* = 8), intrathecal, (*n* = 11), or subcutaneous, (*n* = 7) application together with control IgG subcutaneous (*n* = 5) or intravenous (*n* = 6) PBS. (B) Antibody concentration in CSF 7 days after stroke in animals with intravenous bolus injection of 42 mg anti-Nogo antibody (*n* = 2) on day 2 after stroke *versus* continuous intrathecal application (4.2 mg) via osmotic pump (*n* = 2) starting from day 2 after stroke. (C, D) Antibody concentration in different CNS regions 7 days after stroke depending on intravenous bolus injection (42 mg) on day 2 after insult (C) or continuous intrathecal pump infusion (4.2 mg) starting from day 2 after stroke (D). (E) Table depicting the percentage of antibody reaching the respective tissue region relative to the total amount of applied intravenous or intrathecal antibody. We also calculated the percentage of antibody penetration relative to the total amount of injected antibody for the whole brain wet weight as well as for the spinal cord. Data are presented as mean values of antibody concentrations (μg/ml or μg/g wet weight) measured in serum, CSF, and CNS tissue with SEM. Statistical evaluation was carried out with 1-way (C) ANOVA repeated measure followed by Bonferroni *post hoc*; asterisks indicate significances: ***P < 0.001

ELISA analysis of plasma anti-Nogo A antibody concentrations revealed that antibodies applied intrathecally reached the peripheral circulation from the CNS compartment where they slowly accumulated (Fig. 2A). This is in line with the documented half-life of antibodies in the CSF of 1 to 2 days [10, 11]. Intravenous application of anti-Nogo A antibodies resulted in high values after the first and second bolus injections (Fig. 2A). In contrast, daily subcutaneous injections led to a surprisingly slow increase of the anti-Nogo A antibody levels in the circulation with maximal levels far below those of the intravenous injections (Fig. 2A). This result indicates a poor transfer of the antibody from the subcutaneous space on the back of the animals into the systemic circulation. We then compared anti-Nogo antibody concentrations in the CSF and in different CNS compartments 7 days after stroke after intrathecal (via osmotic pump) or intravenous bolus injection of the antibody. Although the amount of intravenously injected antibody was 10× higher than the intrathecally infused amount, anti-Nogo antibody levels in the CSF were 100× higher in animals with the intrathecal route of administration (Fig. 2B). While a relatively high dose of tissue antibody concentration was measured in the penumbra of animals having received the intravenous bolus injection (40.6 ± 3.6 μg/g wet weight, Fig. 2C), all other parts of the CNS had much lower antibody concentrations (1.9-7.5μg/g wet weight). The proportion of intravenously injected antibodies reaching the CNS (outside of the stroke penumbra) was in the range of 0.001 to 0.004% (Fig. 2E). In contrast to the intravenously injected rats, the animals with intrathecal pumps showed a gradient of antibody tissue concentrations along the spinal cord with the highest values in the lumbar spinal cord (64.8 ± 18.6 μg/g wet weight) and decreasing levels up to the brain (Fig. 2D). For the other brain compartments measured, we found an antibody content about 0.02 to 0.04% of the total intrathecally infused anti-Nogo A antibody (Fig. 2E). For the average whole brain wet weight, we calculated 0.19% of infused antibody penetration (Fig. 2E). Tissue antibody retention after 7 days of intrathecal pump infusion was therefore 3.8× higher in the brain tissue and > 67× higher in the spinal cord than a 10× larger intravenous injection (Fig. 2E).

## Discussion

We find that an acute, 2-week intrathecal application of anti-Nogo A antibodies resulted in a recovery level of skilled forelimb function after a large sensory–motor cortex stroke in adult rats of about 40%, whereas the control groups had poor recovery levels of 10 to 25%. These results are in line with earlier studies where intrathecal control antibodies were compared to anti-Nogo A antibodies [9]. Importantly, no significant improvements of skilled forelimb reaching were observed in the animals injected with anti-Nogo A antibodies intravenously or subcutaneously. The high peripheral antibody levels also did not enhance the damage as shown by comparison with the PBS and the untreated groups. The intrathecal anti-Nogo effect was specific for the trained task—food pellet grasping—underlining the role of training in functional recovery after stroke [6]. In the untrained ladder crossing test, intravenous anti-Nogo-treated rats showed a tendency for a positive outcome, perhaps due to small amounts of antibodies reaching the CNS from the very high plasma levels. Surprisingly, after subcutaneous injections, plasma levels of antibodies rose only very slowly and never reached the levels seen after intravenous injection, in spite of the higher amounts injected. Unspecific, high-capacity binding must have occurred in the subcutaneous compartment with poor uptake and only slow release into the circulation. Therapeutic antibodies targeting CNS antigens are of high current interest for a number of neurological diseases. Easy routes of application would be highly desirable. However, the present data show a clear superiority of application by the intrathecal route directly into the CNS over high doses applied peripherally. We find a relative tissue antibody concentration of ~ 0.5 to 0.8% of the intrathecally infused antibody in the CNS, a concentration that was sufficient to promote fiber rearrangement and functional recovery. Our results are also in line with tissue concentration studies after intrathecal antibody delivery in other animal models [12] as well as in humans [4]. Following intravenous bolus injection, we retrieved 0.007 to 0.05% of the intravenously applied anti-Nogo antibody in the CNS parenchyma. Similar results were found in earlier studies [3, 13, 14]. This proportion may be slightly increased by local inflammatory events, e.g., MS foci or inflamed AD plaque regions in the CNS, which might locally compromise the BBB [3, 15, 16]. Here, however, the large stroke lesions did not provide sufficient BBB breakdown to allow antibody penetration to the extent that the plasticity and repair functions of anti-Nogo A would have become apparent. Several recent clinical trials with intravenously applied therapeutic antibodies failed to reach the expected results, in spite of a strong scientific basis and preclinical results [2, 16, 17]. Poor, individually variable antibody penetration into the CNS may well have played an important role for these failures. Strategies to enhance delivery of systemically administered exogenous antibodies to the CNS such as transient disruption of the BBB with focused ultrasound [18, 19] or shuttling constructs across the BBB [20–22] are being developed but have not stood the tests for clinical routine yet.

Our study reveals that antibody delivery to the CNS remains challenging. While the intrathecal application guarantees defined antibody doses in the effective range for a biological function, strong efforts must be taken to identify and establish easier routes of administration to successfully treat CNS disorders with antibody-based future therapies.

## Electronic supplementary material

ESM 1 (PDF 465 kb)

## References

[1] Freskgard PO, Urich E (2017). Antibody therapies in CNS diseases. Neuropharmacology.

[2] Ferrero J, Williams L, Stella H, Leitermann K, Mikulskis A, O'Gorman J (2016). First-in-human, double-blind, placebo-controlled, single-dose escalation study of aducanumab (BIIB037) in mild-to-moderate Alzheimer’s disease. Alzheimers Dement (N Y).

[3] Ranger A, Ray S, Szak S, Dearth A, Allaire N, Murray R (2018). Anti-LINGO-1 has no detectable immunomodulatory effects in preclinical and phase 1 studies. Neurol Neuroimmunol Neuroinflamm.

[4] Kucher K, Johns D, Maier D, Abel R, Badke A, Baron H (2018). First-in-man intrathecal application of neurite growth-promoting anti-Nogo-A antibodies in acute spinal cord injury. Neurorehabil Neural Repair.

[5] Schwab ME, Strittmatter SM (2014). Nogo limits neural plasticity and recovery from injury. Curr Opin Neurobiol.

[6] Wahl AS, Omlor W, Rubio JC, Chen JL, Zheng H, Schroter A (2014). Neuronal repair. Asynchronous therapy restores motor control by rewiring of the rat corticospinal tract after stroke. Science.

[7] Wahl AS, Erlebach E, Brattoli B, Buchler U, Kaiser J, Ineichen BV, et al. Early reduced behavioral activity induced by large strokes affects the efficiency of enriched environment in rats. J Cereb Blood Flow Metab. 2018:271678X18777661. 10.1177/0271678X18777661 PMC6775586 29768943

[8] Oertle T, van der Haar ME, Bandtlow CE, Robeva A, Burfeind P, Buss A (2003). Nogo-A inhibits neurite outgrowth and cell spreading with three discrete regions. J Neurosci.

[9] Lindau NT, Banninger BJ, Gullo M, Good NA, Bachmann LC, Starkey ML (2014). Rewiring of the corticospinal tract in the adult rat after unilateral stroke and anti-Nogo-A therapy. Brain.

[10] Noguchi Y, Kato M, Ozeki K, Ishigai M (2017). Pharmacokinetics of an intracerebroventricularly administered antibody in rats. MAbs.

[11] Wang Q, Delva L, Weinreb PH, Pepinsky RB, Graham D, Veizaj E, Cheung AE, Chen W, Nestorov I, Rohde E, Caputo R, Kuesters GM, Bohnert T, Gan LS (2018). Monoclonal antibody exposure in rat and cynomolgus monkey cerebrospinal fluid following systemic administration. Fluids Barriers CNS.

[12] Liebscher T, Schnell L, Schnell D, Scholl J, Schneider R, Gullo M (2005). Nogo-A antibody improves regeneration and locomotion of spinal cord-injured rats. Ann Neurol.

[13] St-Amour I, Pare I, Alata W, Coulombe K, Ringuette-Goulet C, Drouin-Ouellet J (2013). Brain bioavailability of human intravenous immunoglobulin and its transport through the murine blood-brain barrier. J Cereb Blood Flow Metab.

[14] Poduslo JF, Curran GL, Berg CT (1994). Macromolecular permeability across the blood-nerve and blood-brain barriers. Proc Natl Acad Sci U S A.

[15] Sevigny J, Chiao P, Bussiere T, Weinreb PH, Williams L, Maier M (2016). The antibody aducanumab reduces Abeta plaques in Alzheimer’s disease. Nature.

[16] Cadavid D, Mellion M, Hupperts R, Edwards KR, Calabresi PA, Drulovic J, et al. Safety and efficacy of opicinumab in patients with relapsing multiple sclerosis (SYNERGY): a randomised, placebo-controlled, phase 2 trial. Lancet Neurol 2019. 10.1016/S1474-4422(19)30137-1 31285147

[17] Meininger V, Genge A, van den Berg LH, Robberecht W, Ludolph A, Chio A (2017). Safety and efficacy of ozanezumab in patients with amyotrophic lateral sclerosis: a randomised, double-blind, placebo-controlled, phase 2 trial. Lancet Neurol.

[18] Carpentier A, Canney M, Vignot A, Reina V, Beccaria K, Horodyckid C (2016). Clinical trial of blood-brain barrier disruption by pulsed ultrasound. Sci Transl Med.

[19] Morse SV, Pouliopoulos AN, Chan TG, Copping MJ, Lin J, Long NJ (2019). Rapid short-pulse ultrasound delivers drugs uniformly across the murine blood-brain barrier with negligible disruption. Radiology.

[20] Yu YJ, Zhang Y, Kenrick M, Hoyte K, Luk W, Lu Y (2011). Boosting brain uptake of a therapeutic antibody by reducing its affinity for a transcytosis target. Sci Transl Med.

[21] Zuchero YJ, Chen X, Bien-Ly N, Bumbaca D, Tong RK, Gao X (2016). Discovery of novel blood-brain barrier targets to enhance brain uptake of therapeutic antibodies. Neuron.

[22] Kanodia JS, Gadkar K, Bumbaca D, Zhang Y, Tong RK, Luk W (2016). Prospective design of anti-transferrin receptor bispecific antibodies for optimal delivery into the human brain. CPT Pharmacometrics Syst Pharmacol.

